# Application of CD54 in diagnosing bone marrow involvement by using flow cytometry in patients with diffuse large B-cell lymphoma

**DOI:** 10.1186/s12885-021-08753-0

**Published:** 2021-09-09

**Authors:** Wei Wang, Yan Li, Xavier Rivera Rivera, Linjun Zhao, Di Mei, Wenqing Hu, Bin Jiang

**Affiliations:** 1 Department of Pathology and Laboratory Medicine, The University of Texas Health Science Center at Houston, 6431 Fannin Street, Houston, Texas USA; 2 Department of Hematology, Peking University International Hospital, Zhong-Guan-Cun Life Science Park Road, Beijing, China; 3 Department of Lymphoma, Peking University International Hospital, Zhong-Guan-Cun Life Science Park Road, Beijing, China

**Keywords:** CD54, Flow cytometry, Diffuse large B-cell lymphoma, Bone marrow involvement

## Abstract

**Background:**

Flow cytometry plays a key role in detecting bone marrow (BM) involvement in patients with diffuse large B-cell lymphoma (DLBCL). To improve its detection sensitivity, we need to explore novel markers. In this study, we detected the expression CD54 on lymphoma cells in BM specimens from DLBCL patients and clarified its diagnostic significance in BM involvement by DLBCL.

**Methods:**

We collected BM specimens from 76 patients with DLBCL (germinal center B-cell (GCB) = 25, non-GCB = 51) and 10 control patients without lymphoma. We detected and compared the expression of CD54 on lymphoma cells and normal mature B cells by using 10-color panels.

**Results:**

Normal plasma cells expressed a higher level of CD54 as compared with hematogones (*p* < 0.05) and normal mature B cells (*p* < 0.05). Among 76 patients, 23 of them (GCB = 12, non-GCB = 11) had BM involvement. Lymphoma B cells from 12 cases (GBC = 4, non-GCB = 8) expressed a higher level of CD54 compared to normal mature B cells (*p* < 0.05). Additionally, lymphoma cells of the non-GCB subtype frequently expressed a higher level of CD54 in comparison to the GCB subtype (*p* < 0.05). And the high expression of CD54 was not related to plasmacytoid differentiation.

**Conclusion:**

Aberrant expression of CD54 on lymphoma cells is frequently seen in patients’ BM specimens involved by DLBCL, especially in the non-GCB subtype. CD54 could be used as a new marker to gate on lymphoma cells and improve the detection sensitivity of BM involvement in patients with DLBCL.

**Supplementary Information:**

The online version contains supplementary material available at 10.1186/s12885-021-08753-0.

## Background

Diffuse large B-cell lymphoma (DLBCL) is a common type of non-Hodgkin lymphoma (NHL) in adults. Around 11–34% of patients have bone marrow (BM) involvement at diagnosis [1–3], which is a crucial parameter for disease staging and prognosis. Flow cytometry has been widely used in the diagnostic evaluation of lymphoma cells for its sensitivity, rapidity, and qualification [2, 4–8]. Diagnosing BM involvement in DLBCL by using flow cytometry relies on the identification of immunoglobin (Ig) light chain restriction and aberrant antigen expression. If the BM infiltrate is extensive, it is easy to diagnose the BM involvement. However, with limited infiltration, the diagnosis is challenging and could be made by setting up the lymphoma gate elaborately with high-quality flow cytometry techniques and rich experiences [7, 9]. Therefore, new markers will add value to improve sensitivity in detecting lymphoma cells.

CD54, intracellular adhesion molecule-1 (ICAM-1) [10], is a surface receptor of the Ig superfamily, which is expressed on various cells, including leukocytes, endothelial cells, and smooth muscle cells [11]. It has five extracellular Ig-like domains, and functions as an adhesive and co-stimulatory molecule [12]. After binding with leukocyte function-association antigen-1, CD54 plays a key role in lymphocyte homing, adhesion, and activation. CD54 also involves in the process of tumor immune response [13, 14] . In this study, we detected CD54 expression on DLBCL lymphoma cells from BM specimens and evaluated its diagnostic significance.

## METHORDS

### Patients

A total of 76 BM specimens from patients with DLBCL were obtained at Peking University International Hospital from 2019 to 2021. All cases were diagnosed as DLBCL according to the latest WHO criteria [15, 16]. There were 46 males and 30 females with a median age of 63 (25–87); 25 were germinal center B-cell (GCB) subtype, and 51 were non-GCB subtype. 60 patients were newly diagnosed, and 16 patients were post-treatment with residual lymphoma cells in the BM confirmed by BM biopsy. At the same time, 10 BM specimens from patients without hematologic malignancies were also enrolled as the control subgroup. The study has been approved by The Hospital Ethical Committee in accordance with the guideline of the Helsinki Declaration of 2008. All clinical and epidemiological data were collected from medical records. General characteristics of patients are shown in Table 1.

**Table 1:** Patients’ basic characteristics

	GCB (*n* = 25)	Non-GCB (*n* = 51)
Gender (Male/Female)	13/12	33/18
Age, median (range)	62 (25–80)	64 (31–87)
Time point (diagnosis/after treatment)	18/7	42/9
Bone marrow involvement (with/without)	12/13	11/40

GCB: germinal center B-cell. The Han’s algorithm was used to classify the GCB subtype and non-GCB subtype

### Flow cytometry

BM specimens were collected in heparin anticoagulant tubes. 5 × 10^5^ white blood cells per tube were stained and analyzed within 8 h after procurement. Flow cytometry analysis was performed on a 4-laser, 10-color Becton Dickinson (BD) FACSCanto™ flow cytometer (San Diego, California, USA). The analyzing software was DIVA (BD).

Multiple fluorescent mouse anti-human monoclonal antibodies (mAbs) were used in the study as listed in Supplementary 1. Kappa, Lambda, and Bcl-2 are from Dako Denmark (Glostrup, Denmark), CD22 is from Thermo Fisher Scientific (Waltham, Massachusetts, USA), FMC-7 is from Beckman Coulter (San Diego, California, USA), and TdT is from Invitrogen (Carlsbad, California, USA). All the other mAbs and Lysing Solution are from BD. The IntraStain kit is from Dako Denmark, which is used to detect the cytoplasmic expression of TdT and Bcl-2. If the expression of surface light chains can’t be detected, we would use the IntraStain kit to permeabilize cells and then detect the cytoplasmic light chains with the similar mAbs cocktail as listed in Supplementary 1.

The gate strategies were set up as follows. In a forward scatter (FSC)/ side scatter (SSC) dot plot, debris, dead cells, and remaining erythrocytes were excluded. Singlets were identified based on FSC-A/FSC-H. Thinking of variable expression of antigens by lymphoma B cells, we used CD19, CD20, and CD45 to set up the mature B-cell gate (CD19++, CD20++, CD45++, and SSC low) in each tube. We also adjusted the mature B-cell gate according to the specific situation (such as high FSC or SSC et al). CD22 and CD79b were used as the complementary markers to confirm the maturity of B cells after the anti-CD20 therapy. CD54 expression was quantified as mean fluorescence intensity ratio (MFIR) (MFI of CD54-BV421/negative control-BV421). The cluster of lymphoma cells should contain at least 20 cells.

### Bone marrow aspirate and biopsy

BM trephine biopsies, aspirate smears, and clot sections were prepared simultaneously. Wright-Giemsa-stained slides of BM aspirate smears were examined. BM tissue samples were fixed and paraffin-embedded. Hematoxylin & eosin (H&E) staining and immunohistochemical (IHC) staining of CD3, CD5, CD10, CD20, CD138, PAX5, MUM-1, Kappa, and Lambda were performed.

### Diagnostic criteria of BM involvement

BM trephine biopsies, aspirate smears, and clot sections were reviewed by two hematopathologists independently. The results were recorded down as positive or negative. If there were disagreements about the diagnosis, we would consult the third hematopathologist. BM biopsy was the diagnostic standard for BM involvement as described previously [17, 18].

### Statistics

The MFIR is presented as mean ± standard error of mean (SEM). The 2-tailed Student’s t-test was used to evaluate the difference of CD54 MFIR between two subgroups. A *p*-value lower than 0.05 is statistically significant. To set up the cut-off value of CD54 MFIR, we used the Receiver Operating Characteristic (ROC) Curve. All the statistical analyses were calculated using GraphPad Prism 6 (GraphPad Software, San Diego, CA, USA).

## Results

Among 76 patients, 18 of them had BM involvement diagnosed by BM biopsy. In the meanwhile, monotypic mature B cells were detected by flow cytometry in the BM specimens of all 18 patients. Additionally, there were 5 patients with monoclonal B cells who were not diagnosed with BM involvement by BM biopsy. Since the immunophenotypic aberrance was so obvious (Supplementary 2), and their BMs were all positive for Ig heavy chain rearrangement detected by PCR, we included them in the BM involvement subgroup during the following analysis.

### CD54 expression profile in normal B-cell

**Fig. 1:**
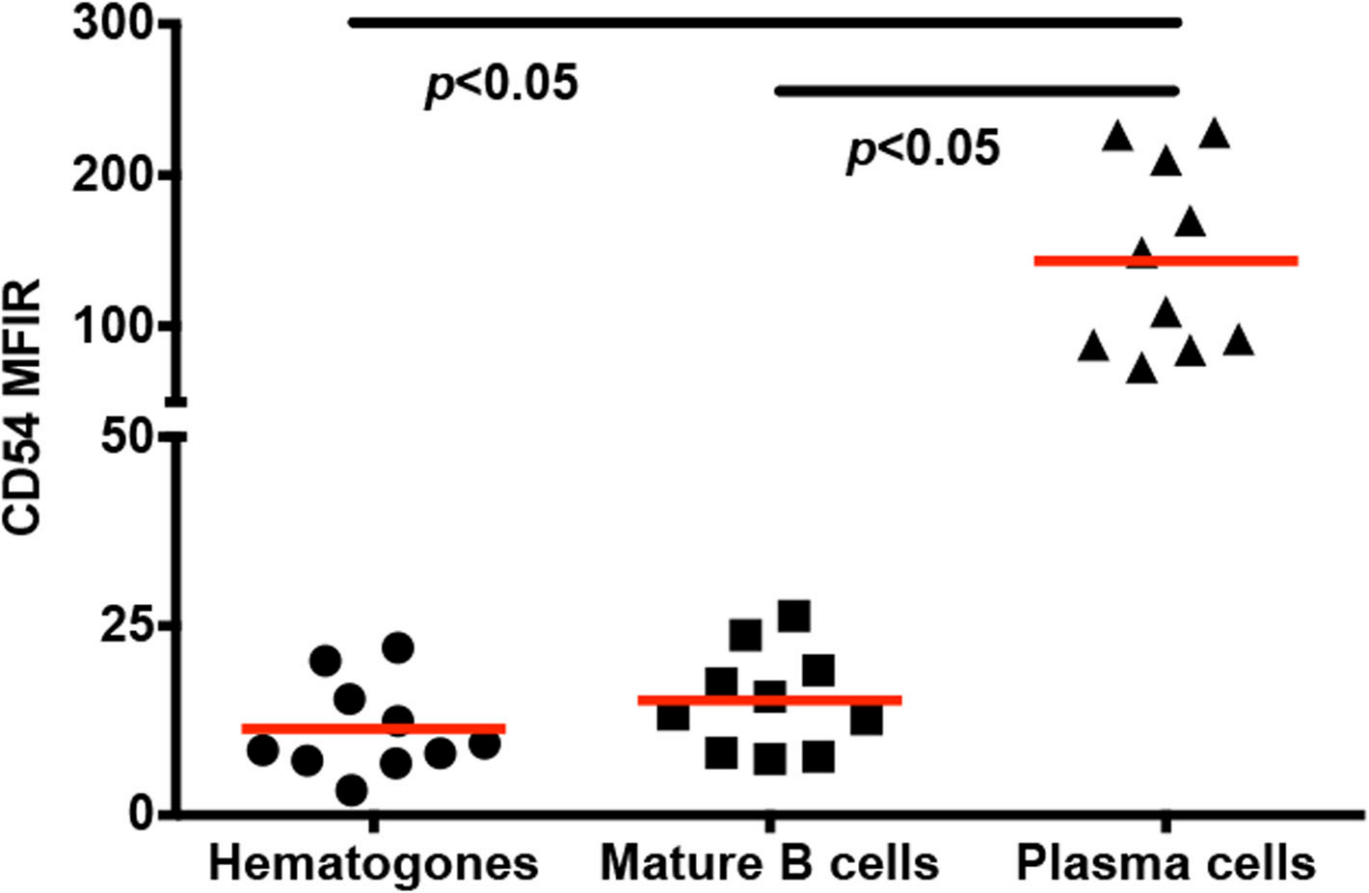
Expression of CD54 in hematogones, mature B cells, and plasma cells. The results of flow cytometry are shown. Among those three populations, plasma cells express a high level of CD54. The 2-tailed Student’s t-test was used. Horizontal bars present the mean value

First, we evaluated the expression profile of CD54 in the control subgroup. We used the following informative markers to discriminate hematogones as previously described [19]: stage 1: CD45+, CD19+, CD34+, TdT+, CD10++, CD38+, CD20-; stage 2: CD45+, CD19+, CD34-, TdT dim/−, CD10+, CD38+, CD20−/dim; stage 3: CD45+, CD19+, CD34-, TdT-, CD10 dim, CD38 variable, CD20 dim/+. Mature B cells were defined as CD45++, CD20++, CD34-, TdT-, and CD10-. While plasma cells were defined as CD45+, CD19+, CD38++, and CD138+. Plasma cells expressed the highest level of CD54 (143.3 ± 19.64, *p* < 0.05) in comparison to hematogones (11.37 ± 1.94) and mature B cells (15.15 ± 2.10). And as for the expression of CD54, there was no difference between hematogones and mature B cells (Fig. 1).

### The expression of CD54 in patients with DLBCL and control subgroups

**Fig. 2:**
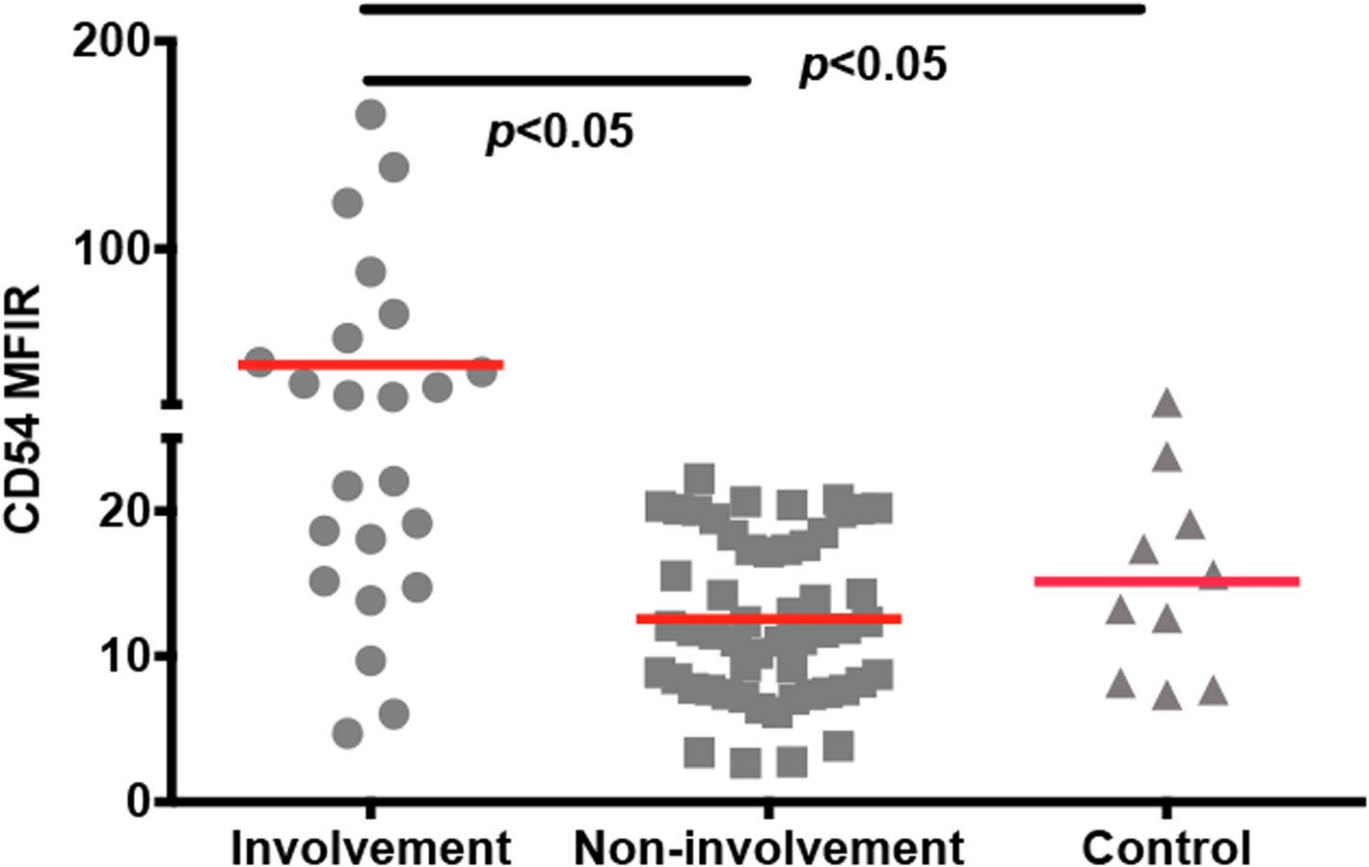
Comparison the expression of CD54 in BM specimens. The results of flow cytometry are shown. We compared CD54 expression in DLBCL patients with and without BM involvement. We also used BM specimens from patients without lymphoma as the control subgroup. Among them, the BM involvement subgroup expresses a high level of CD54. While there is no difference between the non-involvement subgroup and the control one. The 2-tailed Student’s t-test was used. Horizontal bars present the mean value

**Fig. 3:**
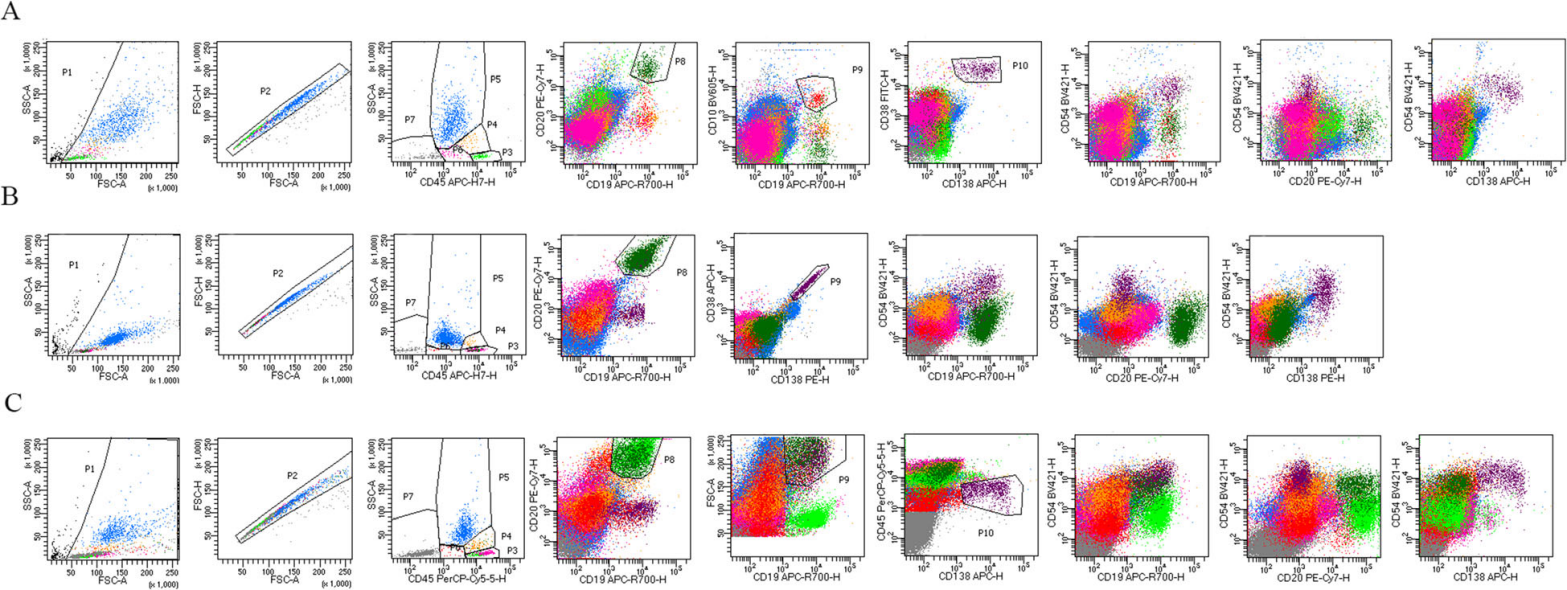
Expression profile of CD54. The results of flow cytometry are shown. A: BM specimen from a patient without lymphoma; B: BM specimen from a DLBCL patient without BM involvement; C: BM specimen from a DLBCL patient with BM involvement. Purple cells: plasma cells; dark green cells: normal mature B cells (A and B) or lymphoma cells (C); light green: normal mature B cells (C). Normal mature B cells express a low level of CD54, while normal plasma cells express a high level of CD54. In Fig. C, lymphoma cells in dark green highly express CD54 compared to the residual normal mature B cells in light green color. These lymphoma cells have large FCS

In this study, the acquired median lymphocytes number was 31,601 (1420 – 123,500), so there were enough lymphocytes to analyze. We separated DLBCL patients into two subgroups, e.g. with or without BM involvement. We also used BM specimens from patients without hematologic malignancies as the control subgroup as mentioned before. We then compared the expression of CD54 in the three subgroups. As shown in Fig. 2, the BM involvement subgroup expressed significantly higher level of CD54 (*n* = 23, 44.24 ± 9.21) in comparison to the non-involvement one (*n* = 53, 12.58 ± 0.76, *p* < 0.05) and the control one (*n* = 10, 15.15 ± 2.10, *p* < 0.05). While there is no difference between the non-involvement subgroup and the control one (*p* > 0.05). Figure 3 shows the expression profile of CD54 from three cases belonging to the three subgroups. Normal mature B cells expressed a low level of CD54. In patients with BM involvement, lymphoma B cells abnormally expressed high levels of CD54. Normal plasma cells could be used as the positive internal control to observed the expression of CD54 on B cells.

Based on the BM biopsy results, 55% (10/18) of cases (5 in GCB, and 5 in non-GCB) have concordant BM involvement as defined previously [17]. Among those 10 cases, flow cytometry detected increased FSC in 8 cases, and 5 of them highly expressed CD54. The other 2 concordant cases having similar FSC as other lymphocytes also expressed high level of CD54. Among those 8 cases with discordant BM involvement, 1 of them highly expressed CD54.

### The expression of CD54 in GCB and non-GCB subtypes with BM involvement

Based on the result of the ROC curve, we set up the cut-off value (27.45, sensitivity 100%, specificity 52.17%) of CD54. In 53 patients without BM involvement, CD54 was all lower than the cut-off value. While in 23 patient with BM involvement, CD54 of 12 patients were higher than the cut-off value.

**Fig. 4:**
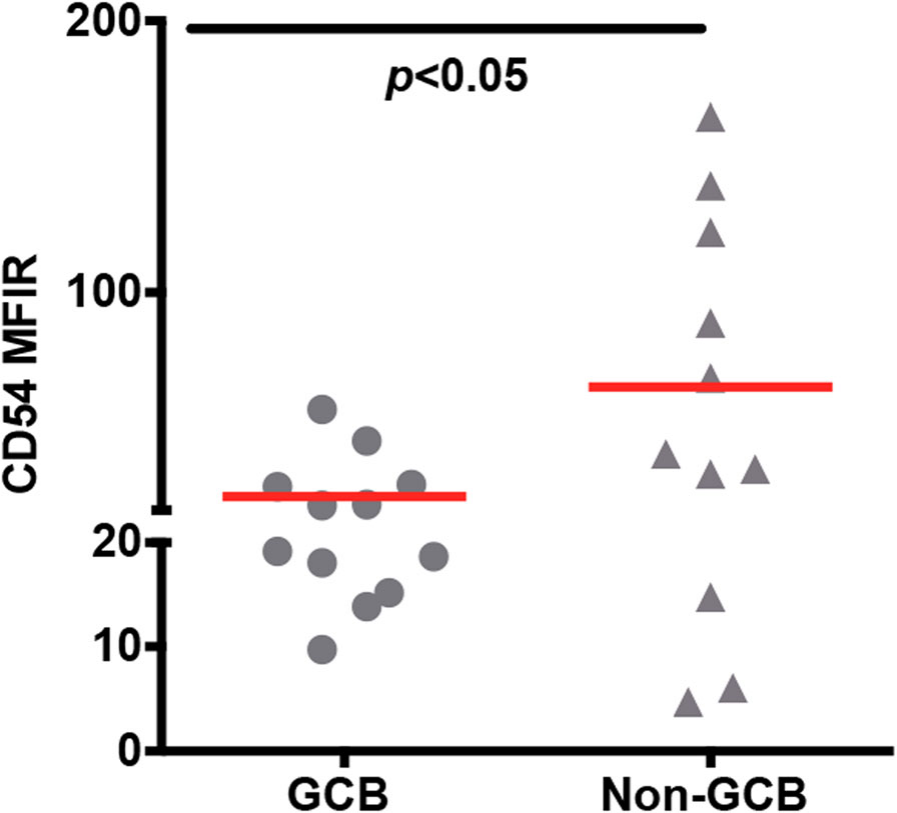
Expression of CD54 in two subtypes of DLBCL with BM involvement. The results of flow cytometry are shown. In DLBCL patients with BM involvement, the non-GCB subtype significantly expresses higher CD54 than the GCB one. The 2-tailed Student’s t-test was used. Horizontal bars present the mean value

Next, we compared the expression of CD54 on lymphoma cells between GCB and non-GCB subtypes with BM involvement. The non-GCB subtype (*n* = 11, 65.32 ± 16.91) presented significantly higher level of CD54 than the GCB one (*n* = 12, 24.90 ± 3.96) (*p* < 0.05) (Fig. 4). On top of that, 72.72% (8/11) of non-GCB patients highly expressed CD54, while 33.33% (4/12) of GCB patients did.

### Immunophenotypic aberrancies of cases with BM involvement

We further evaluated the aberrant antigen expressions in 23 patients with BM involvement. 12 cases (52.17%) showed increased FSC in lymphoma cell clusters. Monoclonal B cells showed in all 23 cases. The other frequently aberrant markers are Bcl-2 (82.61%, 19/23) and CD54 (52.17%, 12/23). In the GCB subtype, abnormal expression of CD10 on lymphoma cells is frequently seen (91.97%, 11/12). CD38 + showed in 30.43% (7/23) of cases, CD25 + in 21.74% (5/23), CD71 + and CD5 + in 8.70% (2/23), CD43+, and CD45 dim in 4.35% (1/23) (Supplementary 3).

### Plasmacytoid differentiation of CD54 high cases

Since normal plasma cells highly express CD54, we then observed whether the abnormal expression of CD54 on lymphoma cells is due to plasmacytoid differentiation, which has been defined previously [20, 21]. As we mentioned before, there were 12 cases highly expressing CD54 on B lymphoma cells. In 8 cases being diagnosed as BM involvement by BM biopsy, there was no clue of plasmacytoid differentiation based on morphology. Their IHC results showed that lymphoma cells were positive for PAX5 and CD20, and negative for CD138 and light chains. While for the other 4 cases whose BM involvement was diagnosed by flow cytometry, their lymphoma cells were positive for CD20, CD22, CD79b, and the surface light chain, while negative for CD138. Thus we conclude that high expression of CD54 on lymphoma cells is not related to plasmacytoid differentiation.

## Discussion

Flow cytometry has been widely proved its diagnostic significance in detecting BM involvement in patients with B-cell non-Hodgkin lymphoma (B-NHL). One of the key points for flow cytometry is to set up an elaborate gating strategy to identify monoclonal B cells as much as possible. Because lymphoma B cells most often show as monoclonal B cells, e.g. B cells with Ig light chain restriction. If the number of infiltrating lymphoma cells is high, with a simple gating strategy (CD45++, CD19+, CD20+), monoclonal lymphoma B cells would show up. On the other hand, aberrant expression of other markers (such as CD10) and light scatter properties are also pivotal parameters to make the diagnosis, which could also be used to set up the gate. However, under the following circumstances, the identification of monoclonal B cells is not straightforward. First, surface Ig is not detectable in some cases of DLBCL [22, 23]. Second, it is very difficult to separate a minimal number of lymphoma cells when mixing up with florid reactive B cells. Third, CD10 is invalid in most cases of the non-GBC subtype. Fourth, the histologic discordance between the BM specimens and lymphoid tissues is not uncommon in B-NHL [10, 24, 25]; thus, the immunophenotype of the primary site (such as lymph node) may not be helpful to set up the lymphoma cell gate. Fifth, the monoclonal B cells do not equal malignancy [26–28]. Sixth, under rare circumstances, if more than one B-cell lymphoma population is present (composite lymphoma or bi-clonal B-cell lymphoma), the underlying lymphoma cells would masquerade as a normal ratio of Ig light chain [29]. To solve these problems and enhance the sensitivity of flow cytometry, we need to explore new markers. Recently, there have been some studies focusing on researching new markers in DLBCL. CD81 is a germinal center marker in both normal mature B cells and DLBCL [30]. Non-GCB DLBCL expresses higher levels of CD39 and CD95 in comparison with follicular lymphoma and Burkitt lymphoma [31].

Previously, there are some studies about the expression of CD54 in patients with B-NHL. Low expression of CD54 on lymphoma cells from primary sites is related to an advanced stage, extranodal involvement, BM infiltration, poor therapeutic response, and worse survival in aggressive B-cell lymphoma [32]. Loss of CD54 on lymphoma cells is related to decreased tumor-infiltrating T cells in patients with DLBCL [33]. In all those studies, the expression of CD54 was detected by using IHC, so it is difficult for us to compare with their results due to different detection methods. Recently, a study using flow cytometry shows that CD54 is significantly higher in DLBCL than Burkitt lymphoma [34]. So far, there are no studies to evaluate the expression profile of CD54 on normal B cells, and the difference of CD54 between normal and lymphoma B cells.

In this research, we clarify that normal hematogones and mature B cells express a low level of CD54. While DLBCL lymphoma cells from 52% of BM specimens highly express CD54. This means that abnormal expression of CD54 on lymphoma cells is a frequent phenomenon in DLBCL patients with BM involvement. In patients with the GCB subtype, CD10 is routinely used to gate on lymphoma cells. However, there is no such marker in the non-GCB subtype. Our research shows that lymphoma cells from around 70% of BMs involved by non-GCB DLBCL abnormally expressed a high level of CD54. Therefore, CD54 is especially useful to detect lymphoma cells in patients with the non-GCB subtype and might be used as a backbone marker to gate on lymphoma cells.

Normal plasma cells highly express CD54. We then clarified that the high expression of CD54 on lymphoma cells is not related to plasmacytoid differentiation. When designing flow cytometry panels, we combined CD54 with markers for plasma cells (e.g. CD38 and CD138) and B cells (e.g. CD19, CD20) in one tube. And we highly recommend combining those markers together to accurately set up the B-cell gate and avoid interference from plasma cells. Since the delay in processing specimens and freeze/thaw could result in the decrease of CD138 expression [35, 36], and the majority of the normal plasma cells are positive for CD19, we highly recommend finishing the detection in time.

In this study, most of the cases with BM involvement were diagnosed by BM biopsy. However, there were 5 cases whose BM involvement was diagnosed by flow cytometry and confirmed by Ig heavy chain rearrangement. As shown in Supplementary 2, the number of lymphoma cells infiltrating BMs might be too low to be detected by BM biopsy (0.03–0.71%). Among them, four cases highly expressed CD54. This also hints that the detection sensitivity of CD54 could attain 0.03%.

In the future, we will enroll more patients, and detect the expression of CD54 in lymphoid nodes, spleen, and other lymphoid tissues. We will also study the prognostic significance of CD54.

## Conclusions

To summarize, lymphoma cells from BM specimens in patients with DLBCL frequently express a high level of CD54. So CD54 could be used as a new gating marker to distinguish lymphoma cells from their normal counterparts after eliminating the disturbance from plasma cells. It is an especially promising marker in the non-GCB subtype.

## Supplementary Information

**Additional file 1: Supplementary 1.** Flow cytometry panels used in this study.

**Additional file 2: Supplementary 2.** Immunophenotype of 5 patients diagnosed as BM involvement by using flow cytometry.

**Additional file 3: Supplementary 3.** Immunophenotypic aberrancies of cases with BM involvement.

## Data Availability

All data generated or analyzed during this study are included in this published article and in its supplementary information files.

## Abbreviations

DLBCL: Diffuse large B-cell lymphoma
NHL: Non-Hodgkin lymphoma
BM: Bone marrow
Ig: Immunoglobin
ICAM-1: Intracellular adhesion molecule-1
GCB: Germinal center B-cell
mAbs: Monoclonal antibodies
FSC: Forward scatter
SSC: Side scatter
MFIR: Mean fluorescence intensity ratio
H&E: Hematoxylin & eosin
IHC: Immunohistochemical
SEM: Standard error of mean
ROC: Receiver Operating Characteristic
B-NHL: B-cell non-Hodgkin lymphoma
