# Supplementary material for: Application of CD54 in diagnosing bone marrow involvement by using flow cytometry in patients with diffuse large B-cell lymphoma
Source: BMC Cancer. 2021 Sep 9;21:1011. doi: 10.1186/s12885-021-08753-0 (PMC8431857; doi:10.1186/s12885-021-08753-0)
Supplement: Supplementary file 1 — Additional file 1: Supplementary 1. Flow cytometry panels used in this study. [file 12885_2021_8753_MOESM1_ESM.docx]

**Supplementary 1. Flow cytometry panels used in this study**

| Panel | BV421 | BV510 | BV605 | FITC | PE | PerCP-Cy5.5 | PE-CY7 | APC | APC-R700 | APC-H7 |
| --- | --- | --- | --- | --- | --- | --- | --- | --- | --- | --- |
| 1 | CD54 | CD34 | CD10 | Bcl-2 | CD38 | CD5 | CD20 | CD138 | CD19 | CD45 |
| 2 | CD138 | CD38 | CD10 | Lambda | Kappa | CD5 | CD43 | CD20 | CD19 | CD45 |
| 3 | CD5 | CD81 | CD23 | FMC7 | CD22 | - | CD79b | CD200 | CD19 | CD45 |
| 4 | CD103 | CD11c | - | TdT | CD30 | CD71 | CD20 | CD25 | CD19 | CD45 |
| 5 | - | CD34 | CD10 | - | CD38 | - | CD20 | CD138 | CD19 | CD45 |
| 6 | CD54 | CD34 | CD10 | TdT | CD38 | - | CD20 | CD138 | CD19 | CD45 |

Panel 1- 4 are used to detect B lymphoma cells. Panel 5 is used as the negative control to calculate the mean fluorescence intensity ratio of CD54. Panel 6 is used only in the control subgroup to clarify the expression profile of CD54 at different maturation stages. If the expression of immunoglobin light chains could not be detected on the surface, cytoplasmic expression of light chains would be detected by using the IntraStain kit and the similar antibody-cocktail of panel 2.
