# Supplementary material for: Application of CD54 in diagnosing bone marrow involvement by using flow cytometry in patients with diffuse large B-cell lymphoma
Source: BMC Cancer. 2021 Sep 9;21:1011. doi: 10.1186/s12885-021-08753-0 (PMC8431857; doi:10.1186/s12885-021-08753-0)
Supplement: Supplementary file 2 — Additional file 2: Supplementary 2. Immunophenotype of 5 patients diagnosed as BM involvement by using flow cytometry. [file 12885_2021_8753_MOESM2_ESM.docx]

**Supplementary 2. Immunophenotype of 5 patients diagnosed as BM involvement by using flow cytometry**

| Number | lymphoma cells/nucleated cells in BM (%) | Subtype | Monotypic subtype | FSC increased | Aberrant antigen expression |
| --- | --- | --- | --- | --- | --- |
| 1 | 0.03 | GCB | Kappa | + | CD54 +  CD71 ++  Bcl-2 + |
| 2 | 0.22 | Non-GCB | Kappa | + | CD54 +  CD71 ++  Bcl-2 + |
| 3 | 0.71 | GCB | Kappa | + | CD10 +  CD38 ++  Bcl-2 + |
| 4 | 0.40 | GCB | Lambda | - | CD10 +  CD54 + |
| 5 | 0.04 | Non-GCB | Kappa | + | CD25 ++  CD54 +  Bcl-2 + |

BM: bone marrow. GCB: geminal center B-cell. FSC: forward scatter.
