# Supplementary material for: Application of CD54 in diagnosing bone marrow involvement by using flow cytometry in patients with diffuse large B-cell lymphoma
Source: BMC Cancer. 2021 Sep 9;21:1011. doi: 10.1186/s12885-021-08753-0 (PMC8431857; doi:10.1186/s12885-021-08753-0)
Supplement: Supplementary file 3 — Additional file 3: Supplementary 3. Immunophenotypic aberrancies of cases with BM involvement. [file 12885_2021_8753_MOESM3_ESM.docx]

**Supplementary 3. Immunophenotypic aberrancies of cases with BM involvement**

|  | GCB (n = 12) | Non-GCB (n = 11) |
| --- | --- | --- |
| Monotypic B cells | 12 | 11 |
| FSC increased | 4 | 8 |
| Aberrant antigens  CD54+  Bcl-2+  CD10+  CD38+  CD25+  CD71+  CD5+  CD43+  CD45dim | 4  9  11  3  1  1  2  0  1 | 8  10  0  4  4  1  0  1  0 |

BM: bone marrow; GCB: geminal center subtype. FSC: forward scatter
